# Risk factors for recurrent wheezing after bronchiolitis in infants: 2-year follow up in China

**DOI:** 10.1186/s12879-021-05937-8

**Published:** 2021-03-10

**Authors:** Sainan Chen, Wenjing Gu, Min Wu, Chuangli Hao, Canhong Zhu, Xuejun Shao, Yuqing Wang

**Affiliations:** 1 Department of Respiratory Medicine, Children’s Hospital of Soochow University, Jingde Road No. 303, Suzhou, 215003 China; 2 Department of Laboratory Medicine, Department of Clinical laboratory, Children’s Hospital of Soochow University, Jingde Road No. 303, Suzhou, 215003 China

**Keywords:** Bronchiolitis, Eczema, Recurrent wheezing

## Abstract

**Background:**

Infants with bronchiolitis have an increased risk of developing recurrent wheezing and asthma. However, the risk factors for the development of recurrent wheezing after bronchiolitis remains controversial. Our study was to investigate risk factors of post-bronchiolitis recurrent wheezing.

**Methods:**

Infants with bronchiolitis were enrolled from November 2016 through March 2017. Nasopharyngeal aspirates were obtained for detection of respiratory viruses which were analyzed by reverse transcriptase polymerase chain reaction (RT-PCR) and direct immunofluorescent assay. Serum cytokines including TSLP, IL2, IL13, TIMP-1, MMP-9, IL33, IL5, IL4, IL25, TNF- α and MIP-1α were measured by flow cytometry. Patients were followed up every 3 months for a duration of 2 years by telephone or at outpatient appointments.

**Results:**

We enrolled 89 infants, of which 81 patients were successfully followed up. In total, 22.2% of patients experienced recurrent wheezing episodes. The proportion of patients with history of eczema, systemic glucocorticoid use and patients with moderate-to-severe disease were significantly higher in the recurrent wheezing group than the non-recurrent wheezing group (83.3% vs 52.4%; 66.7% vs 36.5%; 61.1% vs 33.3%, respectively, all *P* < 0.05); There were no significant differences between patients with and without recurrent wheezing episodes in the levels of TSLP, IL2, IL13, TIMP-1, MMP-9, IL33, IL5, IL4, IL25, TNF- α and MIP-1α (*P* > 0.05). Logistic regression analysis showed that history of eczema was an independent risk factor for post-bronchiolitis recurrent wheezing (odds ratio [OR] = 5.622; 95% confidence interval [CI], 1.3–24.9; *P* = 0.023).

**Conclusion:**

The incidence of recurrent wheezing among infants after contracting bronchiolitis was 22.2% during a 2-year follow-up. History of eczema was the only independent risk factor identified and no correlation was found between the specific virus and disease severity in children with post-bronchiolitis recurrent wheezing.

## Background

Acute bronchiolitis is a common lower respiratory tract disease characterized by inflammation of the bronchioles with a diameter of 75–300 um. It is often caused by viral infection in infants under two years and symptoms include wheezing, cough, tachypnea and chest retractions. Respiratory syncytial virus (RSV) is the most common cause of acute bronchiolitis among infants [1, 2].

Recurrent wheezing is sometimes observed after acute bronchiolitis attacks [3]. Follow up data from abroad showed that the incidence of post-bronchiolitis recurrent wheezing is as high as 31% [4] and about 48% of patients were diagnosed with asthma at the age of 7 years old following severe RSV bronchiolitis [5]. In a follow-up study in China [6], 35.1% (26/74) of infants at 6 months of age or less with bronchiolitis had recurrent wheezing by the age of 3 years. However, the mechanism of recurrent wheezing after bronchiolitis has not been elucidated, and knowledge about risk factors is still controversial.

Current research suggests that risk factors for post-bronchiolitis recurrent wheezing include: allergy [7], the elevation of specific IgE level [8], family history of asthma [9], passive smoking [10], disease severity [11] and others. A large number of epidemiological and prospective studies showed that respiratory syncytial virus infection is a risk factor for recurrent wheezing or asthma after bronchiolitis [5, 12, 13]. Some researchers believe that rhinovirus is a more important factor for recurrent wheezing [14]. In addition, bocavirus and metapneumovirus are also associated with recurrent wheezing and asthma following bronchiolitis [13, 15]. However, Bacharier et al. showed that post-bronchiolitis recurrent wheezing has no relation to viral pathogens [5]. In recent years, increasing attention has been paid to the relationship between cytokine level and post-bronchiolitis recurrent wheezing. One study showed that IL-3 can be used as a predictor of recurrent wheezing after bronchiolitis [16], and some researchers believe that MIP-1 α can be used as a biomarker to predict recurrent wheezing [17]. However, no accurate biomarkers for the prediction of recurrent wheezing are currently available for infants with bronchiolitis.

It is of great significance for treatment and prevention to understand the risk factors for recurrent wheezing following bronchiolitis. However, there are few follow-up studies of bronchiolitis in China, and the risk factors for recurrent wheezing remain unclear. Therefore, the objective of this study was to identify potential predicting factors for post-bronchiolitis recurrent wheezing in infants through a two-year follow-up study.

## Methods

Infants diagnosed with bronchiolitis and hospitalized at the Department of Respiratory Disease at Children’s Hospital Soochow University, China, from November 2016 through March 2017, were enrolled in this cohort study. Nasopharyngeal aspirates were obtained for detecting respiratory virus and analyzed by reverse-transcriptase polymerase chain reaction (RT-PCR) and direct immunofluorescent assay. Serum cytokines including TSLP, IL2, IL13, TIMP-1, MMP-9, IL33, IL5, IL4, IL25, TNF- α and MIP-1α were measured by flow cytometry. The patients were followed every 3 months for a duration of 2 years by telephone or at outpatient appointments.

Recurrent wheezing was defined as two or more episodes following initial bronchiolitis for two years. The study was approved by the ethics committee of the Children’s Hospital Soochow University (Approval No.:2016050). Informed consent was obtained from the parents of all children enrolled in this study.

Inclusion criteria: 1) Age: 1–24 months; 2) Patients were hospitalized with bronchiolitis; 3) Bronchiolitis was defined as the first wheezing episode characterized by cough, tachypnea and chest retractions.

Exclusion criteria: Neuromuscular disease, congenital airway deformity, congenital heart disease, gastroesophageal reflux disease, bronchial foreign body inhalation, primary or secondary immune deficiency or other immune-associated diseases were excluded.

Disease severity criteria: according to Wang expiratory flow limitation (EFL) scoring [18], the severity of disease was graded as follows (Table 1): 0–4.9 was mild; 5–8.9 was moderate; 9–12 was severe.

**Table 1:** Scoring standard of bronchiolitis

	0	1	2	3
R (Times / min)	< 30	31–45	46–60	> 60
Wheezing	No	Only can be heard at the end of expiration by stethoscope	Be heard during expiration with or without stethoscope	Be heard during inspiration and expiration without stethoscope
Tri-retraction sign	No	Only rib gap depression	Trachea depression	Nasal flaring
Mental condition	Normal			Irritability, drowsiness and decreased feeding

### Sample collection

Nasopharyngeal aspirates were obtained using a suction catheter passed through the nose into the lower part of the pharynx for detection of viruses. Peripheral venous blood (2 ml) was also collected for detection of blood routine, humoral and cellular immunity and cytokines.

### Detection of seven common viruses by direct immunofluorescent assay (DFA)

DFA was used to detect respiratory syncytial virus (RSV), influenza virus A (IVA), influenza virus B (IVB), parainfluenza virus (PIV) I, PIV II, PIV III, and adenovirus (ADV). All assay kits were purchased from Chemicon (USA) and all staining procedures were performed according to the manufacturer’s instructions. Immunostained preparations were viewed with a fluorescence microscope (Leica 020–518.500, Germany).

### Detection of the metapneumovirus (hMPV), rhinovirus (hRV), bocavirus (hBoV) gene by real-time polymerase chain reaction (RT-PCR)

For hMPV detection, primers were designed to specifically amplify the N gene (213 base pairs [bps]). The forward and reverse primers were hMPV-F:5′- AACCGTGTACTAAGTGA.

TGCACTC-3′ and hMPV-R:5′- CATTGTTTGACCGGCCCCATAA-3′, respectively. The cyclic temperature settings were 94 °C, 30 s; 55 °C, 30 s; 68 °C, 30 s; amplified by 45 cycles with the last at 68 °C for 7 min.

For hRV detection, the primers and probe sequences were HRV-F: 5′-TGGACAGGGTGTGAAGAGC-3′;HRV-R:5′-CAAAGTAGTCGGTCCCATCC-3′;HRV-probe:FAM-TCCTCCGGCCCCTGA ATG-TAMRA. The cyclic temperature settings were 94 °C, 30 s; 56 °C, 30 s; 72 °C, 30 s; amplified, 40 cycles.

For hBoV detection, the primers and probe sequences were HBoV-F:5′-TGACATTCAACTACCAACAACCTG-3′;HBoV-R:5’CAGATCCTTTTCCTCCTCC.

AATAC-3′; HBoV-probe: FAMAGCACCACAAAACACCTCAGGGG-TAMRA. The cyclic temperature settings were 94 °C,30 s; 56 °C, 30 s; 72 °C, 30 s; amplified for 40 cycles.

### Routine blood test

Peripheral venous blood (2 ml) was collected and anticoagulated with EDTA from every patient. The blood was tested by an automatic five classification hematology analyzer for white blood cell count, absolute neutrophil count, absolute lymphocyte count and absolute count of eosinophils.

### Testing of humoral immunity

Detection indices: IgG, IgM and IgA. IgG and IgM were determined by transmission immunoassay, and IgA was determined by immunoturbidimetry.

### Testing of cellular immunity

Peripheral venous blood (2 ml) was collected on EDTA anticoagulant. Flow cytometry of Beckman Coulter company was used for analysis. The kit was purchased from Immunotech (France). Detection indices: CD3+, CD3+ CD4+, CD4+ / CD8+, CD3 + CD8+, CD19 + CD23+, CD3-CD16 + CD56+ and CD3-CD19 + .

### Detection of serum levels of cytokine

The peripheral blood was centrifuged at 2500 r/min for 5 min. Supernatants were frozen at − 80 °C. Serum levels of cytokines (TNF-α, IL-2, IL-13, IL-4, IL-5, IL-25, IL-33, TSLP, TIMP-1, MMP-9 and MIP-1α) were measured by flow cytometry. Flow cytometry (Beckman Coulter, Brea, CA, USA) was performed according to the manufacturer’s instructions (Immunotech, Marseille, France). All assay kits were purchased from BEIJING TONGSHENG SHIDAI BIOTECH CO., LTD. Data were automatically processed and analyzed using FCAP Array 3.0 with the standard curve produced from the cytokine standard.

### Data collection

Each patient’s data, including age, gender, gestational age at delivery, birth weight, feeding patterns, history of eczema, family history of asthma, exposure to smoking, and pet contact were recorded.

### Follow-up of patients

After discharge from hospital, the patients were followed up every 3 months for a 2-year period by outpatient visits or telephone consultations.

### Statistical analysis

SPSS version 18.0 software was used for data analysis. Distribution normality of continuous data was tested by the P-P plots methods before comparison. Data with normal distribution were represented as mean ± standard deviation (SD) and analyzed by t tests. Continuous data with non-normal distribution were represented as median (minimum-maximum) and analyzed with the Mann-Whitney U test. Categorical data were represented as frequency and analyzed with Chi square examination. Predictors of recurrent wheezing were analyzed using a stepwise logistic regression model.

## Results

### Recruitment and general characteristics

The study included 89 cases (56 males and 33 females; mean age: 4.68 ± 4.37 months).

### Viral detection rate in clinical specimens

The detection rate for respiratory viruses was 50.6%. The most common pathogen was RSV (44.4%), followed by HRV (3.7%) and HBoV (2.5%). Two patients (2.5%) had co-infections with RSV and HRV. (Table 2).

**Table 2:** Virus distribution in the 81 infants with bronchiolitis

Viruses	Total, *n* = 81, no. (%)	RW(−), *n* = 63,no. (%)	RW(+), *n* = 18, no. (%)
RSV	36 (44.4)	29 (47.6)	7 (44.4)
HRV	3 (3.7)	2 (3.2)	1 (5.6)
Metapneumovirus	0 (0)	0 (0)	0 (0)
HBoV	2 (2.5)	1 (1.6)	1 (5.6)
Parainfluenza virus	0 (0)	0 (0)	0 (0)
Influenza virus	0 (0)	0 (0)	0 (0)
Adenovirus	0 (0)	0 (0)	0 (0)
One virus	41 (50.6)	32 (50.8)	9 (50.0)
Two viruses	2 (2.5)	1 (1.6)	1 (5.6)
Not detected	4 (4.9)	2 (3.2)	2 (11.1)

note: RW(+), patients with recurrent wheezing episodes; RW (−), patients without recurrent wheezing episodes

### Follow-up results

**Fig. 1:**
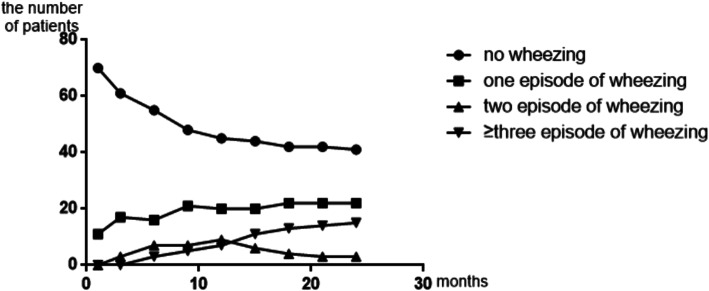
Numbers of wheezing episodes over time in infants with bronchiolitis

A total of 81 patients were successfully followed up and eight patients were lost to follow up due to the wrong telephone number being recorded. Of the 81 infants whose parents answered the telephone interview every three months, 22 (27.2%) infants experienced one single episode and 18 (22.2%) infants experienced recurrent wheezing, including 3(3.7%) with 2 episodes, 15 (18.5%) with ≥3 episodes. (Fig. 1).

### Baseline data between patients with and without recurrent wheezing episodes

History of eczema was more common in patients with recurrent wheezing episodes (*P* = 0.037, continuity correction). There were no significant differences between patients with and without recurrent wheezing episodes with regards to sex, age, premature birth, feeding patterns, family history of asthma and others (*P* > 0.05) (Table 3).

**Table 3:** Comparison of baseline data between patients with and without recurrent wheezing episodes

Factors	RW(−), *n* = 63(%)	RW(+), *n* = 18(%)	statistics	*P*
Sex (male)	41 (65.1)	9 (50.0)	1.348	0.280
Age (months)	5.20 (1.03–19)	3.97 (1.30–9.83)	− 0.704	0.481
premature birth	3 (4.8)	0	0.890	^△^1.000
LBW	4 (6.3)	0	1.202	^△^0.570
Macrosomia	4 (6.3)	2 (11.1)	0.463	^△^0.610
Feeding patterns				
breast feeding	35 (55.6)	11 (61.1)	0.176	0.675
Artificial feeding	7 (11.1)	3 (16.7)	0.051	*0.821
mixed feeding	21 (33.3)	4 (22.2)	0.373	*0.541
eczema	33 (52.4)	15 (83.3)	4.348	*0.037
Family history of asthma	4 (6.3)	2 (11.1)	0.463	^△^0.610
Exposure to smoking	29 (46.0)	9 (50.0)	0.089	^△^0.795
Pet contact	1 (1.6)	1 (5.6)	0.915	^△^0.397

note: RW(+), patients with recurrent wheezing episodes; RW (−), patients without recurrent wheezing episodes; LBW, low birth weight

Data are expressed as % of positive cases, mean ± SD or median (minimum-maximum), unless otherwise stated

* Continuity correction

^△^ Fisher exact test

### Clinical characteristics between patients with and without recurrent wheezing episodes

There were no significant differences between patients with and without recurrent wheezing episodes with regards to fever, feeding difficulty, chest retractions and other clinical characteristics (*P* > 0.05); however, the proportion of patients with moderate to severe conditions in the recurrent wheezing group was higher than that in patients without recurrent wheezing (*P* = 0.033) (Table 4).

**Table 4:** Comparison of clinical characteristics between patients with and without recurrent wheezing episodes

Factors	RW(−), n = 63(%)	RW(+), n = 18(%)	statistics	*P*
Fever	4 (6.3)	3 (16.7)	1.888	^△^0.180
Feeding difficulty	12 (19.0)	5 (27.8)	0.225	*0.636
R > 60 times / min	4 (6.3)	1 (5.6)	0.000	^△^1.000
Chest retractions	3 (4.8)	2 (11.1)	0.974	^△^0.307
Cyanosis	2 (3.2)	2 (11.1)	1.878	^△^0.212
moderate to severe condition	21 (33.3)	11 (61.1)	4.520	0.033

note: RW(+), patients with recurrent wheezing episodes; RW (−), patients without recurrent wheezing episodes; R: respiratory rate

Data are expressed as % of positive cases, unless otherwise stated

* Continuity correction

^△^ Fisher exact test

### Treatment and course of disease between patients with and without recurrent wheezing episodes

Systemic glucocorticoid treatment was higher in patients with recurrent wheezing episodes than those without wheezing episodes (*P* = 0.031, continuity correction). (Systemic glucocorticoid was used during hospitalization for the first attack). There were no significant differences in the need for oxygen treatment, length of hospital stay and the length of illness between children with and without recurrent wheezing episodes (*P* > 0.05) (Table 5).

**Table 5:** Comparison of treatment and courses of disease between patients with and without recurrent wheezing episodes

Factors	RW(−), n = 63(%)	RW(+), n = 18(%)	statistics	*P*
Oxygen inhalation	8 (12.7)	3 (16.7)	0.002	*0.965
Systemic glucocorticoid treatment	23 (36.5)	12 (66.7)	5.189	0.023
Hospital stay(d)	7.63 ± 2.07	8.39 ± 2.09	1.844	0.178
Course of disease(d)	14.97 (10,30)	16.28 (11,30)	−1.400	0.162

note: RW(+), patients with recurrent wheezing episodes; RW (−), patients without recurrent wheezing episodes

Data are expressed as % of positive cases, mean ± SD or median (minimum-maximum), unless otherwise stated

* Continuity correction

### Laboratory examinations between patients with and without recurrent wheezing episodes

There were no significant differences between patients with and without recurrent wheezing episodes in blood cell count, humoral immunity, cellular immunity, serum total Ig E and antigen-specific Ig E (*P* > 0.05) (Table 6).

**Table 6:** Comparison of laboratory examinations between patients with and without recurrent wheezing episodes

Factors	RW(−), *n* = 63(%)	RW(+), n = 18(%)	statistics	*P*
Blood cell count (×10^9^/L)				
White blood cell	9.86 ± 3.78	11.38 ± 4.70	2.017	0.159
Absolute lymphocyte count	6.05 ± 2.84	7.15 ± 2.95	2.061	0.155
Absolute neutrophil count	2.87 (0.24–8.87)	3.04 (0.04–11.49)	−0.358	0.720
Eosinophil count	0.11 (0.00–0.61)	0.09 (0.00–0.59)	− 0.608	0.543
Platelet count	441.41 ± 129.12	454.83 ± 109.95	0.161	0.690
Humoral immunity(g/L)				
IgA	0.18 (0.01–1.03)	0.19 (0.03–0.47)	−0.657	0.511
IgG	4.64 (2.44–10.59)	4.88 (2.76–8.50)	−0.853	0.394
IgM	0.65 ± 0.33	0.67 ± 0.34	0.062	0.804
Cellular immunity				
CD3^+^	0.63 ± 0.10	0.63 ± 0.16	0.011	0.918
CD4^+^	0.42 ± 0.10	0.43 ± 0.11	0.130	0.719
CD8^+^	0.19 ± 0.06	0.18 ± 0.06	0.101	0.752
CD4^+^/CD8^+^	2.52 ± 1.19	2.67 ± 1.26	0.217	0.643
NK	0.13 (0.03–0.38)	0.12 (0.05–0.35)	−0.779	0.436
CD19^+^/CD23^+^	0.12 (0.02–0.31)	0.13 (0.05–0.23)	− 0.087	0.931
Serum total Ig E (IU/mL)	17.13 (0.20–159.50)	38.71 (1.90–367.00)	−0.945	0.345
Antigen-specific Ig E				
Milk protein	4 (6.3)	3 (16.7)	1.888	^△^0.180
Egg protein	2 (3.2)	1 (5.6)	0.223	^△^0.535

note: RW(+), patients with recurrent wheezing episodes; RW (−), patients without recurrent wheezing episodes

Data are expressed as % of positive cases, mean ± SD or median (minimum-maximum), unless otherwise stated

* Continuity correction

^△^ Fisher exact test

### Serum cyctokines in patients with and without recurrent wheezing episodes

There were no significant differences between patients with and without recurrent wheezing episodes in the levels of TSLP, IL2, IL13, TIMP-1, MMP-9, IL33, IL5, IL4, IL25, TNF- α and MIP-1α (*P* > 0.05) (Table 7).

**Table 7:** Comparison of serum level of cytokines between patients with and without recurrent wheezing episodes

cytokines	RW(−), n = 63	RW(+), n = 18	statistics	*P*
IL-2	4.43 (1.25–17.86)	3.87 (1.17–6.78)	−0.357	0.721
TNF-α	1.18 (0.00–4.57)	0.78 (0.00–2.97)	−0.410	0.159
IL-4	25.02 (6.86–47.18)	20.81 (7.83–39.7)	−1.609	0.108
IL-5	7.52 (2.41–16.06)	6.50 (1.78–11.55)	−1.021	0.307
IL-13	3.01 (0.75–6.42)	2.52 (1.22–4.20)	− 1.392	0.164
MIP-1α	14.25 (7.68–32.24)	12.98 (7.61–18.85)	−0.825	0.409
IL-25	60.11 (19.53–124.22)	48.46 (15.99–108.74)	−1.833	0.067
IL-33	30.84 (12.69–77.18)	27.93 (7.75–50.12)	−0.643	0.520
TSLP	10.34 (3.98–18.42)	9.01 (3.27–14.16)	−1.259	0.208
MMP-9	1575.09 (23.71–9040.32)	1436.08 (116.00–6946.29)	−0.825	0.409
TIMP-1	379.44 (207.97–881.72)	427.50 (256.60–797.00)	−1.343	0.179

note: RW(+), patients with recurrent wheezing episodes; RW (−), patients without recurrent wheezing episodes

Data are expressed as median (minimum-maximum), unless otherwise stated

### Analysis of risk factors of recurrent wheezing after bronchiolitis

We used single factor analysis to evaluate the statistically significant or near significant variables (history of eczema, systemic glucocorticoid treatment, moderate to severe condition, IL-25). By logistic multivariable regression analysis, history of eczema was identified as potential risk factor for recurrent wheezing with an OR (95% CI) of 5.622 (1.3,24.9); *P* < 0.05 (Table 8).

**Table 8:** Logistic multivariable regression of the risk factors

Predictive variable	*P*	OR	95% CI
Eczema	0.023	5.622	1.3–24.9
Systemic glucocorticoid treatment	0.053	3.883	1.0–15.4
Moderate to severe condition	0.366	1.927	0.5–8.0
IL-25	0.470	0.989	1.0–1.0

## Discussion

In this study, 49.4% of the infants had at least one wheezing episode, and 22.2% of infants had experienced recurrent wheezing during the 2-year follow-up period. Several previous studies reported that the incidence of recurrent wheezing in infants with bronchiolitis was about 16–60% [3–6, 17, 19–22]. Zhang X et al. [6] followed up 74 Chinese infants who were hospitalized for bronchiolitis until the age of 3 years and found that 35.1% of the infants had recurrent wheezing. Another 1.5-year follow-up study [20] showed the prevalence of post-bronchiolitis recurrent wheezing among infants was 19.4%. In previous studies, allergy, antigen-specific Ig E, family history of asthma, exposure to smoking, severity of disease and specific pathogens or serum level of cytokines were found to be risk factors for recurrent wheezing [7, 9–11, 13].

Asthmatic diseases often occur in individuals with allergic or atopic constitution, and allergic diseases may have similar pathogenesis [23]. Children with allergic constitution are more susceptible to severe RSV infection and have a higher risk of developing airway hyperresponsiveness [24]. In a case-control study [7], it was found that 48% of children had recurrent wheezing, and allergic constitution was considered to be a risk factor. It has been reported that allergy has the strongest correlation with eczema before the age of 1 year old [25]. Several studies have shown that recurrent wheezing in infants is related to allergic dermatitis (eczema) [26, 27]. Dumas et al. [28] analyzed the prospective data of 921 hospitalized children with bronchiolitis in 17 centers in the United States, and found that the risk of recurrent wheezing was significantly increased in children with eczema. It has been confirmed that eosinophils play a key role in the pathogenesis and development of atopic diseases including asthma and eczema [29, 30]. Accordingly, one study [31] reported that recurrent wheezing at 36 months after bronchiolitis in infants was associated with eosinophilia. In our study, the proportion of infants with history of eczema in the recurrent wheezing group was 83.3%, which was significantly higher than in the non-recurrent wheezing group. Multivariate regression analysis suggested that history of eczema was the only independent risk factor for recurrent wheezing post bronchiolitis. Therefore, it is suggested that early intervention should be carried out in infants with bronchiolitis accompanied by eczema in order to reduce the incidence of recurrent wheezing.

Several prior studies have reported that RSV infection in early life may have a profound impact on the development of recurrent wheezing and/or asthma [5, 13, 32]. Other studies demonstrated that hRV-related bronchiolitis may be associated with an increased prevalence of recurrent wheezing and asthma in later childhood [9, 33]. However, studies by teeratakulpisan et al. [19] and Valkonen et al. [34] found no consistent relationship between the type of virus and recurrent wheezing. In our study, 44.4% of infants were infected with RSV and 3.7% were infected with hRV. The RSV rate was lower with respect to the above studies which may be related to the test method (DFA) being associated with variable and lower sensitivity compared with PCR. We found there was no significant difference in terms of the causative virus and recurrent wheezing after bronchiolitis. As such, we suggest that further study is required to ascertain whether type of virus can be used as a predictor of recurrent wheezing.

Two demographically and clinically important differences were observed in our study between the two groups, namely, the percentage of infants with moderate to severe bronchiolitis and the usage of systemic glucocorticoids were significantly higher in infants with recurrent wheezing. A longitudinal study [11] found that in 343 children with bronchiolitis caused by RSV, the severity of the disease could be used as a predictor of asthma and atopic diseases. In addition, a 7-year follow-up study [35] showed that prednisolone could reduce recurrent wheezing after the first rhinovirus infection while another study [36] showed that dexamethasone could not reduce post-bronchiolitis recurrent wheezing 1 year later. Our study did not find that severe bronchiolitis or the usage of systemic glucocorticoids was a risk factor for recurrent wheezing, which is different from other studies. This may be related to the small sample size and/or the inclusion of less severe cases.

The pathogenesis of bronchiolitis is mainly related to excessive type 2 and/or deficient type 1 immune responses [37]. Plasma level of TNF-α, which originates from Th1 cytokine, has been reported to be of great significance in predicting the development of recurrent wheezing during acute bronchiolitis [38]. The serum level of IL-3, IL-4, IL-10, and IL-13, which originate from Th2 cytokine, were higher in children with RSV bronchiolitis secondary wheezing, and IL-3 can be used as a predictor of recurrent wheezing [16]. In our study, multivariate regression analysis showed that there was no significant difference in serum cytokine levels between patients with and without recurrent wheezing episodes. This may be related to the fact that we tested serum samples rather than nasopharynx aspirates.

## Conclusions

In conclusion, the current study indicates the following conclusions: 1. the incidence of recurrent wheezing among infants after bronchiolitis was 22.2% across a 2-year follow-up; 2. history of eczema is a significant risk factor for post-bronchiolitis recurrent wheezing; 3. no correlation was found between the specific virus and disease severity in children with post-bronchiolitis recurrent wheezing.

Our study has some limitations. Firstly, the sample size was small, and the follow-up time was relatively short. Secondly, we could not confirm whether parents gave reliable information in the phone interview. Thirdly, pathogen detection was not performed for every wheezing attack following bronchiolitis. Lastly, immunofluorescence assays have variable and lower sensitivity compared with PCR which may lead to a lower virus detection rate.

## Data Availability

The datasets used and/or analysed during the current study available from the corresponding author on reasonable request.

## Abbreviations

OR: odds ratio
CI: confidence interval
RSV: respiratory syncytial virus
IVA: influenza virus A
IVB: influenza virus B
PIV: parainfluenza virus
ADV: adenovirus
hMPV: metapneumovirus
hRV: human rhinovirus
hBoV: human bocavirus
SD: standard deviation
RW: recurrent wheezing
